# Assessment of the impacts of ergonomic risk factors on shopping centre employees

**DOI:** 10.2478/aiht-2023-74-3741

**Published:** 2023-12-29

**Authors:** Abdullah Köse, Ömer Gündoğdu

**Affiliations:** Igdir University, Graduate Education Institute, Igdir, Turkey; Ataturk University, Mechanical Engineering Department, Erzurum, Turkey

**Keywords:** psychological risks, safety, workplace ergonomics scale, workplace social environment, društveno okruženje na radnom mjestu, ljestvica ergonomije na radnom mjestu, psihološki rizici, sigurnost

## Abstract

Employees of shopping malls face various physiological and psychological health problems due to their specific working conditions. The purpose of this descriptive study was to evaluate the ergonomic risk factors for employees (N=222) from three shopping centres in the city of Erzurum, Turkey. We wanted to learn more about the attitudes of the shopping mall employees regarding their current working conditions, which we hoped would help us propose measures for the elimination or reduction of the most prominent ergonomic risk factors. Data were collected between May 1 and July 1, 2022 using our own questionnaire, which consisted of questions regarding personal characteristics (gender, age, education level, marital status, working year, unit, working position, nature of the job, presence of chronic disease, etc.) and the Workplace Ergonomics Scale, which consists of 32 items divided into 6 sub-dimensions (Occupational Health and Safety, Environmental Conditions, Psychological Elements, Employee Safety, Workplace Social Environment, and Working Environment). The obtained results indicated that the majority of employees were young, healthy, high school graduates mainly working as sales consultants. Their average income was low, their working hours were long, and they found their rest periods shorter than needed. The study found that, although shopping centre employees rated their work environment as low risk in terms of ergonomics, their scores on the workplace ergonomics scale were above average. The results of this study could contribute to a better understanding and identification of ergonomic risks in the trade sector and serve for planning future prevention strategies.

## Abstract (sl)

Zaposlenici trgovačkih centara suočeni su s raznim fiziološkim i psihološkim poteškoćama zbog specifičnih uvjeta njihova posla. Svrha ovoga istraživanja bila je procijeniti ergonomske faktore rizika za zaposlenike (N=222) triju trgovačkih centara u gradu Erzurum, Turska. Namjera nam je bila prikupiti podatke o stavovima zaposlenika spram njihovih trenutačnih uvjeta na radu, koji bi mogli poslužiti za osmišljavanje mjera otklanjanja ili umanjenja najistaknutijih ergonomskih faktora rizika. Podaci su prikupljani u razdoblju od 1. svibnja do 1. srpnja 2022. koristeći samostalno kreirani upitnik, koji je sadržavao pitanja vezana uz osobne značajke (spol, dob, razina obrazovanja, bračni status, godina zaposlenja, jedinica, radno mjesto, priroda posla, kronične bolesti, itd.) i ljestvicu ergonomije na radnom mjestu, koja se sastoji od 32 stavke podijeljene u šest poddimenzija (zdravlje i sigurnost na radu, okolišni uvjeti, psihološki čimbenici, sigurnost zaposlenika, društveno okruženje na radnom mjestu i radno okruženje). Rezultati pokazuju da je većina zaposlenika bila mlađe dobi, odličnog zdravstvenog stanja, imala srednjoškolsku razinu obrazovanja i radila kao prodavači. Njihov je prosječni prihod bio nizak, radno vrijeme dugo, a vrijeme dozvoljeno za odmor smatrali su prekratkim. Naše istraživanje je pokazalo da, iako su ispitanici ocijenili svoje radno okruženje niskorizičnim u smislu ergonomije, njihovi rezultati na ljestvici bili su natprosječno visoki. Rezultati ovoga istraživanja mogli bi pridonijeti boljem razumijevanja i definiranju ergonomskih rizika u sektoru trgovine i poslužiti za planiranje boljih strategija za njihovu prevenciju.

The primary objective of ergonomics is to maximize human-system interactions by considering the relationships between human anatomy, physiology, biology, and psychology, as well as the work environment and equipment. This dynamic discipline continues to evolve as novel insights into human physiology become available. The continual development of technology has given rise to a number of advancements and improvements, like working with displays and robotics, which have caused concerns for workers. Concerns like these may lead to challenges, decreased productivity, and lower quality. The goal of ergonomics is to solve these problems and raise the workers’ productivity-quality ratio (1, 2, 3, 4, 5, 6).

While shopping centres are places that meet a wide range of needs, including the cultural and social needs of their customers, with facilities such as restaurants, cafes, clothing stores, markets, hairdressers, tailors, children's entertainment centres and car parks, they also contain many occupational health and safety problems for their employees.

The number of shopping centres is increasing rapidly all over the world and in Turkey as well (7). While shopping malls generally provide temporary employment to unqualified workers during the construction phase, after they are built, they can provide employment opportunities mainly to women and young workers due to the working principles of the retail industry (8).

Being closed spaces, mostly artificial lighting, mechanical air-conditioning, noisy, complex and large-scale structures, shopping centres (malls) represent a work environment with many challenges and risks for the employees who, by spending most of the day in these places, cannot benefit from the sunlight sufficiently. Mall employees may face with serious physiological and psychological health problems (9). In order to prevent and minimize the occurrence of these health problems, it is important that the ergonomic conditions of shopping malls are suitable for employees (10).

Terece (unpublished PhD thesis) examined the physical comfort criteria in a shopping centre in Istanbul. The study found that the shopping centre had appropriate natural and artificial lighting, and that the wide glass ceiling offered sufficient daylight. The study also emphasized that this situation is important for people with partial visual impairment.

Akyıldız (11), investigated the interior lighting of Antalya shopping centres. By examining window illumination, the author discovered that the brightness level was higher than required, resulting in higher electricity expenses and needless carbon emissions. His conclusion was that Antalya malls do not follow international lighting standards. Therefore, it was suggested that artificial lighting be emphasized in university courses, and that alternative energy sources with reduced energy consumption should be considered.

Kumaş (12) determined that satisfaction with the lighting in the Trabzon Forum Mall was low. Within the scope of the study, six socio-demographic questions and 18 questions regarding user opinions were asked. From the answers given, it was concluded that colour and light should be used proportionally in visual illuminations. It was also argued that more attention should be paid to lighting because it affects human mental well-being.

Kılıç and Tuluç (13) evaluated the noise pollution in open shopping malls in the Kocaeli Province of Turkey. Noise measurements were made in different parts of the shopping mall. It was found that the highest noise was in the children's playground and in the amphitheatre in the evening. In order to prevent this situation, it is recommended to perform afforestation between departments. Again, the idea of putting noise curtains around the car park, which can be considered noisy, was expressed.

İldeş et al. (9), conducted a survey to gather data on the effects of indoor comfort conditions on shopping centre employees in the Edirne Province. In the study, satisfaction was observed regarding thermal comfort conditions in general. However, some other negative effects such as lighting, bad smell, and noise were also detected and certain ergonomic risks were mentioned.

When Shang et al. (14) evaluated the results of the survey in four different shopping malls in China, they drew attention to volatile organic compound concentrations and paid particular attention to the possibility of the symptoms of sick building syndrome in employees.

Through a survey they administered to 1093 automotive and textile employees in the Turkish Bursa Province, Polat et al. (15) developed a workplace ergonomics scale, by considering occupational health and safety issues to measure the effect of ergonomics factors.

Extensive literature research has shown that shopping centres themselves are mostly examined in terms of ergonomic risks, but the ergonomic risk factors of their employees are investigated to a smaller extent.

To add new information relevant for the research field, we designed a research that explored the effects of ergonomic risk factors in shopping centre employees in Erzurum Province (Turkey) by focusing on several issues, adopted from an earlier study by Polat et al. (15). Our main research questions were as follows: (1) What are the socio-demographic characteristics of shopping centre employees? (2) What are the characteristics of shopping centre employees regarding their educational status? (3) Do the socio-demographic characteristics of the shopping centre employees affect the Workplace Ergonomics Scale mean scores? (4) Does the educational status of the shopping centre employees affect the Workplace Ergonomics Scale mean scores? (5) Is there a relationship between the income status of shopping centre employees and their Workplace Ergonomics Scale scores? (6) Do the health characteristics of shopping centre employees affect the Workplace Ergonomics Scale mean scores?

To the best of the authors’ knowledge, no previous study investigated how ergonomic risk factors affect the employees of shopping centres in Erzurum. In this context, the outcomes of our study could help employers in these shopping centres determine their current situation and provide their employees with the appropriate conditions, as well as to eliminate or minimize ergonomic risk factors in line with the results found. Providing suitable working conditions will also contribute to the formation of healthy working environments and increase work efficiency in the long run.

## MATERIALS AND METHODS

### Study design

This research is a descriptive study, which was conducted between May 1 and July 1, 2022. The participants of the study were the employees of three shopping centres. We examined how age, gender, workplace, and other demographic data of individuals working in the shopping centres affect their workplace ergonomics perceptions.

The research was carried out with the approval of the Scientific Research and Publication Ethics Committee of Iğdır University, dated April 12, 2022, no. 2022/6. All survey participants answered the questions by signing informed consent forms.

### Sample

The universe of the research consisted of 300 employees working in the selected shopping centres. The sample selected for the study consisted of 222 subjects who worked there between the specified dates and agreed to participate in the research.

### Data Collection and Analysis

For the collection of research data, we used 1) our own structured interview questionnaire (“Descriptive Characteristics Form”) discussing the characteristic of the subjects and 2) “Workplace Ergonomics Scale” developed by Polat et al. (15).

Between May 1 and July 1, 2022, after the necessary explanations were given to the shopping centre employees about the study, the survey and scale forms were applied to the employees who agreed to participate. The application time of the data collection tools took an average of 15–20 minutes.

The questionnaire consisted of a total of 12 questions, developed in line with the relevant literature, questioning the introductory characteristics of the employees (gender, age, education level, marital status, working year, unit, working position, nature of the job, presence of chronic disease, etc.).

The Workplace Ergonomics Scale we used was a 5-point Likert type scale originally proposed by Polat et al. (15) and consisted of 32 items divided into 6 sub-dimensions (Occupational Health and Safety, Environmental Conditions, Psychological Elements, Employee Safety, Workplace Social Environment, and Working Environment). The scale was obtained by sampling 1093 employees. The Cronbach's Alpha value of the scale was found to be 0.932. The lowest score that can be obtained from the scale is 32 and the highest 160 (15).

The collected data were entered into SPSS 25 (Statistical Package for Social Sciences, IBM, Erzurum, Turkey) software, and appropriate statistical analyses (descriptive statistics, one-way ANOVA, Mann Whitney U-test, t-test, and Kruskal Wallis test) were performed. With Tukey analysis, the workplace ergonomics scale was analysed according to the education level of shopping mall employees. Games-Howell analysis was used in the workplace ergonomics scale according to the work position of the shopping centre employees. Comparison of workplace ergonomics scale total and sub-dimension mean scores of shopping mall employees according to the nature of the job they had was also made with Tukey analysis. The level of significance was set to p<0.05.

## RESULTS AND DISCUSSION

### Personal characteristics; demographic items

The average age of the employees was 28.09±5.52, with 44.6 % subjects between the ages of 26 and 32. Among them, 51.4 % were women, 70.7 % were single, whereas 93.7 % did not have a chronic disease and 77.9 % did not have any health complaints (Table 1).

**Table 1:** Personal and professional characteristics of the shopping centre employees (N=222)

**Characteristics**		**N**	**%**
Gender	Woman	114	51.40
	Man	108	48.60
Marital status	Married	65	29.30
	Single	157	70.70
Age (years) (Mean±SD: 28.09±5.52 years) (Range: 18–54 years)	18–25	85	38.30
	26–32	99	44.60
	33–40	32	14.40
	≥41	6	2.70
Education	Primary school	15	6.80
	High school	87	39.20
	Associate degree	55	24.80
	Undergraduate	60	27.00
	Graduate	5	2.30
Working position	Security guard	25	11.30
	Cleaning staff	24	10.80
	Sales consultant	105	47.30
	Food	25	11.30
	Administration	43	19.40
Nature of work	Requires confidentiality	21	9.50
	Requires attention	143	64.40
	Does not require much attention and confidentiality	36	16.20
	Other	22	9.90
Presence of chronic disease	Yes	14	6.30
	No	208	93.70
Health complaint	Yes	49	22.10
	No	173	77.90
Income (Mean±SD: 240.41±71.14 USD) (Range: 45.60–560.62 USD)	0–107	31	14.00
	108–215	9	4.10
	216–322	165	74.30
	323–430	15	6.80
	431–590	2	0.90
Duration of employment (years) (Mean±SD: 3.74±2.87 years) (Range: 1 month–20 years)	<1 year	34	15.30
	1–5 years	140	63.10
	6–9 years	36	16.20
	10–15 years	9	4.10
	≥16 years	3	1.40
Daily working time (h) (Mean±SD: 9.26±2.19 h) (Range: 2–18 h)	2–6 h	10	4.50
	7–10 h	145	65.30
	11–16 h	67	30.20
Daily rest time (h) (Mean±SD: 9.52±6.62 h) (Range: 1–48 h)	1–5 h	62	27.90
	6–10 h	91	41.00
	10–15 h	40	18.00
	≥16 h	29	13.10

### Work characteristics

The majority of the employees were high school graduates, and 47.3 % of them worked as sales consultants.

Their working period was 3.74±2.87 years on average, and 63.1 % of them worked between 1 and 5 years. It was determined that the average daily working hours were 9.26±2.19 and 65.3 % of them worked 7–10 hours a day. As many as 64.4 % of subjects worked in a job that required a higher level of attention. In addition, it was determined that the daily required rest period was 9.52±6.62 hours on average, and 41.0 % of them needed 6–10 hours of rest per day (Table 1).

The average monthly income of the shopping centre employees included in the research at that time was 240.41± 71.14 USD, and 74.3 % of them were paid in the range of 214.65–321.89 USD. The overall evaluation of the working conditions and income status of the employees showed that their average income was low, working hours long, and rest periods shorter than needed.

### Ergonomic risk factors

It was determined that the total mean score of the workplace ergonomics scale of the shopping centre employees was 113.64±25.21.

Considering the workplace ergonomics scale sub-dimensions of the shopping centre employees, the Occupational Health and Occupational Safety sub-dimension mean score was 25.60±6.89, the Environmental Conditions sub-dimension mean score 20.82±6.24, the Psychological Factors sub-dimension mean score 20.40±3.94, the Occupational Safety sub-dimension mean score 21.05±6.61, the Workplace Social Environment sub-dimension mean score 12.90±4.81, and the Work Environment sub-dimension mean score 12.84±4.26 (Table 2).

**Table 2:** Workplace Ergonomics Scale and the sub-dimension mean scores of shopping centre employees (N=222)

	**Mean±SD**	**Min**	**Max**
Workplace Ergonomics Scale total score	113.64±25.21	32	160
Occupational health and safety	25.60±6.89	7	35
Environmental conditions	20.82 ±6.24	6	30
Psychological elements	20.40±3.94	5	25
Employee safety	21.05±6.61	6	30
Workplace social environment	12.90±4.81	4	20
Working environment	12.84±4.26	4	20

High scores on the Workplace Ergonomics Scale indicate low risk and low scores indicate high risk. Accordingly, when the employees evaluated their workplaces in terms of ergonomic risk factors, low risk was observed, the lowest risk in the Occupational Health and Occupational Safety dimension and the highest risk in the Workplace Social Environment and Working Environment dimensions. The data from this study are compatible with the results of the research conducted by Doğru and Çakır (16) involving employees of an advertising agency.

The comparison of the total and sub-dimension point averages of the workplace ergonomics scale according to the descriptive characteristics of the shopping centre employees is given in Table 3.

**Table 3:** Comparison of Workplace Ergonomics Scale sub-dimensions and total scores according to the descriptive characteristics of the shopping centre employees (N=222)

**Introductory Features**		**Occupational Health and Safety**	**Environmental Conditions**	**Psychological Elements**	**Employee Safety**	**Workplace Social Environment**	**Working Environment**	**Workplace Ergonomics Scale Total Score**
		**Mean±SD**	**Mean±SD**	**Mean±SD**	**Mean±SD**	**Mean±SD**	**Mean±SD**	**Mean±SD**
Gender	Woman	26.07±6.68	21.28±6.27	20.85±3.55	21.42±6.60	13.03±4.94	12.88±4.31	115.56±25.97
	Man	25.12±7.11	20.34±6.21	19.92±4.28	20.66±6.64	12.76±4.69	12.80±4.23	111.62±24.34
**Test/significance**		U/p:5599.000/0.241	t/p:-1.119/0.246	U/p:5400.500/0.110	U/p:5691.500/0.330	t/p:-0.411/0.681	t/p:-0.140/0.889	t/p:-1.162/0.246
Marital status	Married	26.26±6.17	21.15±5.76	20.04±3.69	21.89±6.22	14.01±4.14	13.40±3.84	116.76±21.72
	Single	25.33±7.17	20.68±6,44	20.55±4.04	20.71±6.76	12.44±5,00	12.61±4.41	112.35±26.48
**Test/significance**		U/p:4899.500/0.639	t/p:0.505/0.200	U/p:4510.000/0.168	U/p:4476.500/0.149	**t/p:2.412/0.017**	t/p:1.245/0.215	t/p:1.288/0.200
Education level	Primary Education	21.60±7.48	17.26±5.31	17.00±5.15	19.33±6.19	13.00±3.58	12.33±3.67	100.53±26.30
	High School	26.04±7.47	21.18±6.19	20.83±3.72	21.70±6.49	13.42±4.39	13.47±3.97	116.66±23.63
	Associate Degree	25.36±6.56	21.14±6.22	20.89±3.82	20.72±6.89	12.78±5.51	12.52±4.47	113.43±26.22
	Licence	26.30±6.10	21.63±5.98	20.50±3.40	21.18±6.47	12.45±4.96	12.73±4.52	114.80±25.06
	Graduate	24.40±5.12	12.00±4.84	16.60±5.45	17.20±8.75	10.40±5.36	8.40±2.96	89.00±23.03
**Test/significance**		KW/P:6.567/0.161	**F/P:4.315/0.002**	**KW/P:13.645/.009**	KW/P:3.672/0.452	F/p:0.733/0.570	F/p:2.002/0.095	**F/p:2.628/0.035**
Working position	Security Guard	18.44±3.12	19.08±3.35	18.44±3.12	21.44±4.19	13.80±3.47	13.68±3.32	110.48±15.62
	Cleaning Staff	19.25±4.02	20.62±4.89	19.25±4.02	19.16±6.69	12.83±3.37	12.20±3.65	108,75±16,95
	Sales Consultant	21.11±3.37	20.83±6.66	21.11±3.37	21.40±6.15	12.54±5.05	12.88±4.03	115.00±25.51
	Food Industry	20.28±4.15	20.64±6.34	20.28±4.15	18.24±8.23	12.64±5.55	11.80±5.37	107.56±27.47
	Executive	20.53±4.99	22.02±7.03	20.53±4.99	22.69±7.31	13.46±5.17	13.23±4,88	118.44±30.58
**Test/significance**		KW/P:8.140/0.087	F/p:0.89/0.469	**KW/P:16.094/0.003**	**KW/P:10.308/0.036**	F/p:0.525/0.717	F/p:0.837/0.503	F/p:1.158/0.330
Nature of work	Requires Confidentiality	26.23±7.69	18.90±7.23	20.14±5.17	21.00±7.68	12.47±5.14	11.28±4.30	110.04±31.73
	Requires Attention	25.68±6.74	21.32±5.90	19.93±3.89	21.62±5.91	13.26±4.38	13.37±3.89	115.20±23.49
	Does not Require Much Attention and Confidentiality	23.36±6.21	20.19±5.43	21.27±3.45	17.13±7.88	10.86±5.47	11.05±5.03	103.88±23.60
	Requires Great Attention and Confidentiality	28.18±7.51	20.45±8.35	22.31±2.93	23.86±5.25	14.31±5.33	13.81±4.30	122.95±28.05
**Test/significance**		**KW/P:8.935/0.030**	F/p:1.113/0.345	**KW/P:10.762/0.013**	**KW/P:13.665/0.003**	**F/p:3.210/0.024**	**F/p:4.361/0.005**	**F/p:3.214/0.024**
Presence of chronic disease	Available	23.78±8.21	17.35±7.29	20.42±6.64	16.07±8.00	9.21±5.89	9.92±5.49	96.78±32.51
	Absent	25.73±6.80	21.05±6.12	20.40±3.71	21.39±6.39	13.15±4.64	13.04±4.11	114.78±24.32
**Test/significance**		U/p:1280,000/0.447	**t/p:-2.163/0.032**	U/p:1180.000/0.230	**U/p:861.000/0.010**	t**/p:-3.017/0.003**	t/p:-2.081/0.056	**t/p:-2.619/0.009**
Health complaint	Available	23.69±7.47	16.42±5.69	20.06±4.51	17.87±6.07	9.02±4.17	10.42±3.57	97.51±20.85
	Absent	26.15±6.64	22.06±5.83	20.50±3.77	21.95±6.50	14.00±4.40	13.53±4.20	118.21±24.50
**Test/significance**		**U/p:3428,500/0.040**	**t/p:-6.006/0.000**	U/p:4115,000/0.753	**U/p:2609,500/0.000**	**t/p:-7.071/0.000**	**t/p:-4.705/0.000**	**t/p:-5.387/0.000**

^*^KW refers to the Kruskal-Wallis test (used to test whether the means of two or more samples show a significant difference from each other).

^*^U refers to the Mann-Whitney U test (non-parametric test that is an alternative to the independent sample t test).

^*^F represents the f value (harmonic mean of precision and sensitivity).

^*^t refers to the t test (used to test whether there was a statistically significant difference between two independent groups by looking at the means).

^*^p value indicates whether the relationship was significant or not.

^*^The Mann-Whitney U test with “u” was used to examine the mean difference between two independent groups from a similar population and to determine the difference or equality between groups

We did not find a statistically significant difference between the total and sub-dimension mean scores of the workplace ergonomics scale according to the gender of the shopping mall employees. However, when the total and sub-dimension mean scores of the workplace ergonomics scale were compared according to marital status, it was found that the Workplace Social Environment sub-dimension mean scores showed a statistically significant difference (p<0.05). The “Workplace Social Environment Sub-Dimension” refers to the social environment opportunities where employees can spend their break times. The average score of the Workplace Social Environment sub-dimension of married shopping centre employees was higher than that of single shopping centre employees (Table 3).

When the total and sub-dimension mean scores of the workplace ergonomics scale were compared according to the education level of the shopping mall employees, it was determined that there was a statistically significant difference between the mean scores of the environmental conditions sub-dimension, the psychological factors sub-dimension, and the workplace ergonomics scale (p<0.05).

Evaluation of data using Tukey's test showed that the difference between the environmental conditions sub-dimension and psychological factors sub-dimension, and the total scores of the workplace ergonomics scale according to the education level of the shopping mall employees stemmed from employees with a graduate education level. Employees with graduate education had lower environmental conditions sub-dimension, psychological factors sub-dimension, and workplace ergonomics scale total scores than employees with high school, an associate degree or undergraduate education, and they consider the environmental conditions, psychological factors, and workplace ergonomics in their working environments to be riskier (Table 3).

When the total and sub-dimension mean scores of the workplace ergonomics scale were compared according to the position they work in, it was determined that there was a statistically significant difference between the mean scores of the psychological factors sub-dimension and the worker safety sub-dimension (p<0.05). Using the Games-Howell analysis, it was determined that the difference between the psychological factors sub-dimension scores of the shopping centre employees according to the position they work in is due to those who work as security guards and those who work as sales consultants; that the mean score of the psychological factors sub-dimension of employees working as security guards is statistically significantly lower than those working as sales consultants; and security officers found the psychological elements in their working environments more risky in terms of ergonomics (Table 3).

With regard to the low psychological factors, the following comments can be made. Psychological and ergonomic sub-dimensions scores are low for achieving the satisfaction of the customers they encounter as sales consultants, and also because of employers’ attitudes such as “the customer is always right”, due to fear of making a mistake and losing the customer. However, security guards have a high risk in terms of both psychological and ergonomics due to situations such as standing and constantly moving during almost their entire workday, encountering more people than sales consultants and dealing with them individually. This situation causes the sub-dimension score average to be low due to the psychological factors involved in the work done by the security guards.

Using the Games-Howell analysis, it was found that the difference between the employee safety sub-dimension scores of the shopping centre employees according to the position they work in was caused by those working in the food service and those working in managerial positions, the employee safety sub-dimension mean score of employees in the food sector was statistically significantly lower than those working as managers, and the employees in the food sector found the work conditions of the workers in their working environments more ergonomically risky (Table 3). This situation can be interpreted as follows: employees in the food service may have low scores in terms of psychological factors because they expect the same satisfaction response from all of them, although they know that they first encounter dissatisfied customers and that not all customers will experience the same level of satisfaction. Employees in managerial positions have higher scores than those working in the food service in terms of psychological factors, as they usually encounter customers who are angry because of their dissatisfaction later than other employees, and because an angry customer tends to calm down a little more by the time they have a chance to speak to them. In addition, the environment in the food service is in higher risk in terms of ergonomics, as it contains more dangers than the environment in which the managers are located.

When the total and sub-dimension mean scores of the workplace ergonomics scale were compared according to the nature of the work of the shopping centre employees, it was found that there was a statistical difference between the mean scores of Occupational Health and Work Safety, Psychological Aspects, Employee Safety, Workplace Social Environment, Work Environment, and Workplace Ergonomics Scale Total Scores. It was determined that there was a significant difference (p<0.05).

The difference observed after Tukey's analysis arises from the group working jobs that require more attention and confidentiality, and when the total and sub-dimension point averages of the workplace ergonomics scale of the employees in this group are compared, it was determined that they found it more risk-free in terms of ergonomics (Table 3).

When the total and sub-dimension mean scores of the workplace ergonomics scale according to the presence of chronic disease of the shopping centre employees were compared, it was determined that there was a statistically significant difference between the mean of environmental conditions, Employee Safety, and Workplace Social Environment sub-dimensions and Workplace Ergonomics Scale Total Scores (p<0.05). It was determined that the environmental conditions, Occupational Safety, and Workplace Social Environment sub-dimensions mean scores and the Workplace Ergonomics Scale Total Scores of employees with chronic illness were statistically significantly lower than those without chronic illness. It was also determined that employees with chronic diseases found the ergonomics of their workplaces riskier than employees with no chronic diseases. The fact that places such as shopping malls are very crowded and ventilation cannot be done properly, as well as the fact that they have to share the same space with people who may have different diseases, affect those with chronic diseases more than those without chronic diseases. This makes shopping centres risky for those with chronic diseases in terms of ergonomic, environmental, employee, and psychological factors.

When the total and sub-dimension mean scores of the workplace ergonomics scale were compared according to the presence of health complaints of the shopping mall employees, it was found that there was a statistical difference between the mean scores of Occupational Health and Work Safety, environmental conditions, Worker Safety, Workplace Social Environment, Work Environment, and Workplace Ergonomics Scale. It was determined that there was a significant difference (p<0.05) and that the Occupational Health and Occupational Safety, Environmental Conditions, Occupational Safety, Workplace Social Environment, Work Environment sub-dimensions and Workplace Ergonomics Scale Total Score averages of employees with health complaints were statistically significantly lower than those without health complaints. It was determined that employees with health complaints find the ergonomics of their workplaces riskier than employees without health complaints. People with health complaints are more sensitive than healthy individuals. As all kinds of factors may exist in their environment that could adversely affect their health, this affected them more and caused them to describe their workplace ergonomics as being marked by high risk.

A weak positive and statistically significant relationship between the income status of the shopping mall employees participating in the research and the Occupational Health and Safety, Occupational Safety, and Working Environment sub-dimensions and the total scores of the Workplace Ergonomics Scale was observed. The sub-dimensions of the workplace environment and the total scores of the workplace ergonomics scale also increased (p<0.001, Table 4). There was a weak and negative statistically significant relationship between the working time of the shopping mall employees participating in the research and the mean score of the Occupational Safety and Working Environment sub-dimension. As the working time increased, the Occupational Safety and Working Environment sub-dimension score averages decreased. There was a weak, negative and statistically significant, relationship between daily working hours and the Occupational Safety, Workplace Social Environment, and Work Environment sub-dimension mean scores and Workplace Ergonomics Scale Total Scores. As the working hours increased, the Occupational Safety, Workplace Social Environment, and Work Environment sub-dimension mean scores and Workplace Ergonomics Scale Total Scores decreased.

**Table 4:** The relationship between personal and occupational characteristics of shopping centre employees and the sub-dimension of the Workplace Ergonomics Scale and total scores (N=222)

		**Occupational** **Health and Safety**	**Environmental Conditions**	**Psychological Elements**	**Employee Safety**	**Workplace Social Environment**	**Working Environment**	**Workplace Ergonomics Scale Total Score**
Income	r	0.160^*^	.109	−.065	.142^*^	.127	.090	.137^*^
	p	**0.017**	.105	.338	**.035**	.060	.180	**.041**
Operation time	r	0.010	−.041	−.079	−.187^**^	−.129	−.192^**^	−.126
	p	0.881	.546	.242	**.005**	.054	**.004**	.061
Daily working hours	r	−0.031	−.105	−.098	−.180^**^	−.182^**^	−.224^**^	−.170^*^
	p	0.650	.118	.146	**.007**	**.006**	**.001**	**.011**
Rest time	r	0.064	−.108	−.011	.013	.011	−.091	−.021
	p	0.344	.107	.873	.846	.870	.179	.757
Age	r	0-.009	−.075	−.075	−.060	.029	−.052	−.052
	p	0.892	.264	.264	.376	.665	.443	.443

* Correlation is significant at the 0.05 level.

** Correlation is significant at the 0.01 level

As the working time (year) increased, the increase in the self-confidence of the employees led to weaker compliance with the rules that had to be followed at the workplace. This caused a negative relationship between the working time of the employee and the safety of the employee and the working environment (p<0.001, Table 4). The prolongation of daily working hours caused mental fatigue, distraction, anger, unhappiness, and restlessness among the employees. In such situations, employees exhibited more unsafe behaviours and made their environments more dangerous. In addition, this created an ergonomically negative workplace environment. All this in turn caused the prolongation of daily working hours to be negatively related to employee safety, workplace social environment, working environment, and workplace ergonomics.

There was no statistically significant relationship between the number of hours of rest and average age needed by the shopping centre employees participating in the research and the mean scores of the Workplace Ergonomics Scale and its sub-dimensions (Table 4).

Our study has some limitations as well. Due to the large number of items in the scale, the time required to fill out the data collection forms, and the busy schedules of the employees, the entire population could not be reached, and hence approximately 74 % of the population was covered. The results of the research can be generalized to the shopping centre employees in the entire province.

## CONCLUSION

The research findings indicate that shopping centre employees perceive their workplaces as having low ergonomic risk factors. Employees specifically assessed their workplaces as exhibiting the most danger in terms of the working environment and workplace social environment, and the lowest risk in terms of occupational health and safety. High employee motivation, willingness to learn, enhanced productivity, and a strong sense of care for their work have all been linked to psychological satisfaction. The use of appropriate tools and equipment in accordance with occupational health and safety standards, regular tool and equipment replacement, and employee involvement were all associated with higher levels of satisfaction with occupational safety.

The study also showed that people who are married, have less education, and no chronic illnesses or health issues view their working environments as less ergonomically safe than people who are single, have more education, and have chronic illnesses or health issues. Additionally, workers at shopping malls believe their jobs are less hazardous in terms of ergonomics when their income level rises. Finally, the study reveals that workers at shopping centres believe that when their working hours increase in number, so does the ergonomic risk at work.

We hope that the results of this study could contribute to a better understanding and identification of ergonomic risks in the trade sector and serve for planning future prevention strategies.
